# Comprehensive dissection into morpho-physiologic responses, ionomic homeostasis, and transcriptomic profiling reveals the systematic resistance of allotetraploid rapeseed to salinity

**DOI:** 10.1186/s12870-020-02734-4

**Published:** 2020-11-24

**Authors:** Ying-na Feng, Jia-qian Cui, Ting Zhou, Ying Liu, Cai-peng Yue, Jin-yong Huang, Ying-peng Hua

**Affiliations:** School of Agricultural Sciences, Zhengzhou University, Zhengzhou, 450001 China

**Keywords:** Allotetraploid rapeseed, Differential gene expression, Ion homeostasis, Morpho-physiologic response, Salinity resistance

## Abstract

**Background:**

Salinity severely inhibit crop growth, yield, and quality worldwide. Allotetraploid rapeseed (*Brassica napus* L.), a major glycophyte oil crop, is susceptible to salinity. Understanding the physiological and molecular strategies of rapeseed salinity resistance is a promising and cost-effective strategy for developing highly resistant cultivars.

**Results:**

First, early leaf senescence was identified and root system growth was inhibited in rapeseed plants under severe salinity conditions. Electron microscopic analysis revealed that 200 mM NaCl induced fewer leaf trichomes and stoma, cell plasmolysis, and chloroplast degradation. Primary and secondary metabolite assays showed that salinity led to an obviously increased anthocyanin, osmoregulatory substances, abscisic acid, jasmonic acid, pectin, cellulose, reactive oxygen species, and antioxidant activity, and resulted in markedly decreased photosynthetic pigments, indoleacetic acid, cytokinin, gibberellin, and lignin. ICP-MS assisted ionomics showed that salinity significantly constrained the absorption of essential elements, including the nitrogen, phosphorus, potassium, calcium, magnesium, iron, mangnese, copper, zinc, and boron nutrients, and induced the increase in the sodium/potassium ratio. Genome-wide transcriptomics revealed that the differentially expressed genes were involved mainly in photosynthesis, stimulus response, hormone signal biosynthesis/transduction, and nutrient transport under salinity.

**Conclusions:**

The high-resolution salt-responsive gene expression profiling helped the efficient characterization of central members regulating plant salinity resistance. These findings might enhance integrated comprehensive understanding of the morpho-physiologic and molecular responses to salinity and provide elite genetic resources for the genetic modification of salinity-resistant crop species.

**Supplementary Information:**

The online version contains supplementary material available at 10.1186/s12870-020-02734-4.

## Background

Soil salinization is a serious limiting factor worldwide for high productivity and high quality of agriculture crops [1, 2]. Approximately 50% of arable lands are thought to be detrimentally affected by salinity, and this proportion is estimated to increase continuously, due to the emergence of global extreme climate and overuse of hyper-saline irrigation water [3–5]. Soil salinity inhibits crop growth and development, which in turn reduces yield production, through a two-phase physiological dysfunction: (i) osmotic stresses that declines water potential and (ii) ion toxicity that disturbs ion homeostasis [6]. These stresses are associated with the disorder of a variety of biological processes, including cellular homeostasis imbalance, oxidative stress, essential nutrient dysfunction, protein synthesis disruption, retarded organ growth, and even plant death [7].

In higher plants, maintaining a low Na^+^ level and a suitable range of K^+^/Na^+^ ratios in cells is necessary to enhance plant salinity resistance [6, 8]. Overaccumulation of excessive Na^+^ in the cytoplasm is prevented using three main strategies in plants: (i) inhibiting Na^+^ influx into cytoplasma, (ii) elevating Na^+^ sequestration into vacuoles, and (iii) enhancing Na^+^ efflux from intracellular parts [9, 10]. Under salinity stress, some non-selective cation channels (NSCCs), K^+^ permeases, and other type transporters allow Na^+^ influx into plant cells [11]. Thus, maintaining a suitable ion homeostasis is an important strategy for the resistance of plants to salinity. *Salt Overly Sensitive* (*SOS*) signaling pathways and *non-selective cation channels* (*NSCCs*) are key to the regulation of root Na^+^ influx and efflux [12, 13]. Na^+^/H^+^ antiporters (NHXs) mediate the transport of Na^+^ into the vacuole, and contribute to vacuolar Na^+^ compartmentation that is crucial for the plant SSR. Besides, enhancing the biosynthesis of compatible osmolytes, antioxidants, and polyamines, and maintaining homeostasis of reactive oxygen species (ROS) and phytohormones are also pivotal strategies for plants resistant to salinity stress [14].

Allotetraploid rapeseed (*Brassica napus* L.) is widely used for production of edible vegetable oil, livestock protein meal, and industrial biodiesel [15]. The allotetraploid rapeseed (A_n_A_n_C_n_C_n_, ~ 1345 Mb, 2*n* = 4*x* = 38) is derived from its diploid progenitors *B. rapa* (A_r_A_r_, ~ 485 Mb, 2*n* = 2*x* = 20) [16] and *B. oleracea* (C_o_C_o_, ~ 630 Mb, 2*n* = 2*x* = 18) [17–19]). The rapeseed genome contains numerous duplicated chromosomal segments and homeologous genomic regions, further resulting in polygenic family formation [18, 20].

Salinity greatly hinders the rapeseed biomass and seed yield [20]. Despite various publications on the improvement of rapeseed salinity resistance, limited progress has been made in this direction [21–23]. A comprehensive understanding of the morpho-physiologic and molecular mechanisms underlying the salinity resistance may facilitate the improvement in crop performance under salinity. Breeding and deployment of the salt-resistant rapeseed genotypes having good performance can be an appropriate solution to maintain an optimal yield under salinity [24]. Morpho-physiologic responses of rapeseed plants to salinity were identified and genome-wide transcriptional profile of rapeseed seedlings in response to salinity was investigated in the present study. This study might enrich understanding of the morpho-physiological strategies involving the resistance of rapeseed to salinity, and the identification of core salt-responsive differentially expressed genes (DEGs) might provide elite genetic resources for molecular breeding of salinity-resistant rapeseed germplasm.

## Results

### Morphologic responses of oilseed rape to salinity

**Fig. 1:**
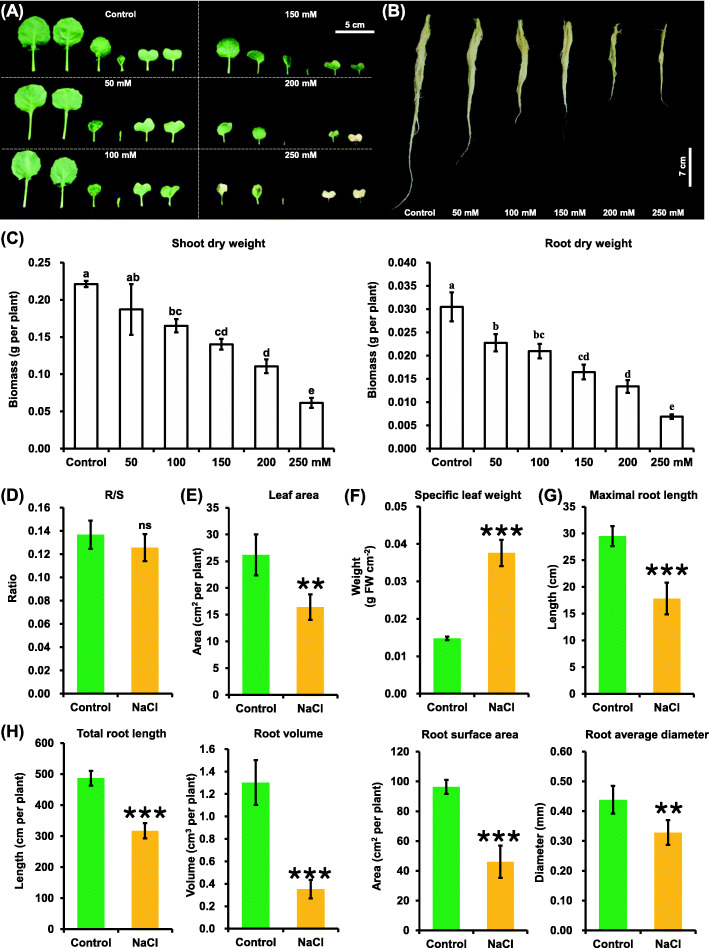
Growth performance of rapeseed plants under the control and salinity conditions. (A-B) Growth performance of the shoots (**a**) and roots (**b**) in rapeseed plants under different dosages of salt conditions. **c** Shoot and root dry weight of the rapeseed plants under different NaCl concentrations. The uniform rapeseed plants after 7-day seed germination were grown under NaCl-free (control) for 10 days, and then were changed to the solution containing 0–250 mM NaCl for 5 days. Different lowercases indicate the significant differences at *P* < 0.05. (D-H) Root/shoot (R/S) ratio (**d**), leaf area (**e**), specific leaf weight (**f**), maximal root length (**g**), and root system architecture (**h**) under control and 200 mM NaCl conditions. For **d**-**h**, uniform rapeseed plants after 7-day seed germination were grown under NaCl-free (control) for 10 days, and then they were transferred to the solution containing 200 mM NaCl for 5 day. Data are means (±SD), *n* = 5. ns, not significant; *, *P* < 0.05; **, *P* < 0.01; ***, *P* < 0.001

Rapeseed plants were grown in hydroponic solution under 0 (control), 50 mM, 100 mM, 150 mM, 200 mM, and 250 mM NaCl conditions to select the most suitable NaCl concentrations for the rapeseed salinity resistance study. Compared with the control condition, the rapeseed plants started to show obvious growth retardation, including leaf necrosis and root inhibition, when the NaCl concentrations were higher than 100 mM (Fig. 1a, b). The salinity-induced growth repression was also indicated by a significant decrease in the shoot and root biomasses (Fig. 1c). The reduction in the shoot and root dry weight was up to 50% with 200 mM NaCl (Fig. 1c), which was used in the following salinity experiments and was also widely applied in previous studies [25].

In this study, no significant changes were observed in the root/shoot ratio with 200 mM NaCl (Fig. 1d). Subsequently, the specific effect of salinity on the shoot and root growth was determined (Fig. 1e-h). Salinity caused a decrease of 40% in total leaf areas (Fig. 1e). However, specific leaf weight was doubled under salinity (Fig. 1f), indicating a marked increase in the leaf thickness. On the contrary, salinity led to a significant reduction in various root system architecture-related parameters, including the maximum length (Fig. 1g), total length, volume, surface area, and average diameter (Fig. 1h).

**Fig. 2:**
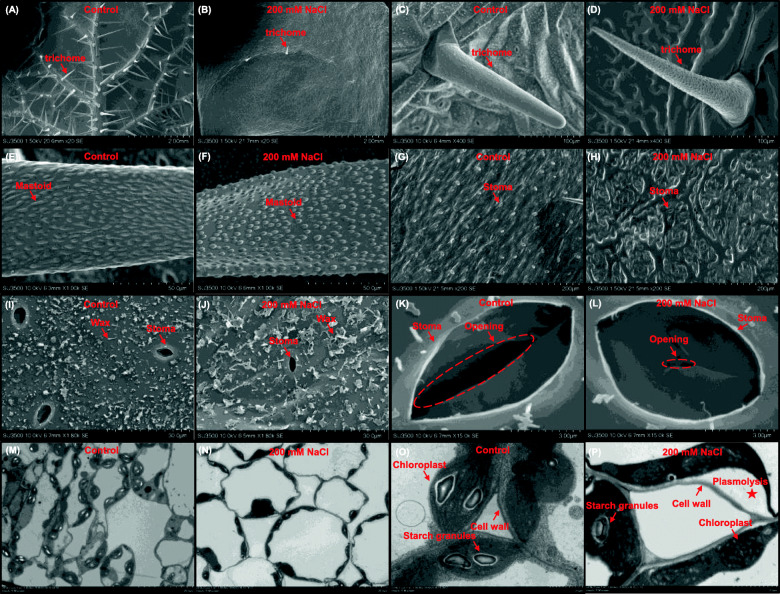
Microscopy characterization of leaf ultrastructure of rapeseed plants under the control and salt conditions. **a**-**d** Low-magnification view (**a**, **b**) and close-up images (**c**, **d**) of leaf trichomes. (**e**-**f**) Mastoid morphology on the surface of trichomes. **g**-**j** Low-magnification view (**g**, **h**) and morphology of wax coats on leaf surfaces (**i**, **j**). **k**-**l** Close-up images of stoma. **m**-**p** Low-magnification (**m**, **n**) and close-up (**o**, **p**) view of chloroplasts along plasma membranes and cell morphologies. Uniform rapeseed plants after 7-day seed germination were cultivated under NaCl-free (control) for 10 days, and then they were transferred to the solution containing 200 mM NaCl for 5 days. The trichomes, mastoids, stoma, cell walls, chloroplasts, and starch granules are indicated by arrows, and the plasmolysis is denoted by an asterisk. The opening of the stoma is indicated by dashed circles

A scanning electron microscope was used to observe trichome and stoma morphology of young leaf epidermis and transmission electron microscope was used to identify organelle damages, so as to examine the intracellular ultrastructure underlying the morphological differences under the control and salt conditions (Fig. 2). The number of leaf trichomes significantly was reduced under salinity compared with the control condition (Fig. 2a, b). However, the overall morphology and surface mastoids of the leaf trichomes were not significantly altered under the control and salinity conditions (Fig. 2c, f). The stoma number obviously reduced under salinity than under the control condition (Fig. 2g and h). In addition, the wax coat of leaf surface was found to be larger under salinity than under the control condition (Fig. 2i, j). Moreover, the stoma showed a higher closure degree under salinity than under the control condition (Fig. 2k, l). The number of starch granules in the chloroplasts markedly decreased under salinity, which also resulted in detached cells and shrunken plasma membranes, namely plasmolysis (Fig. 2m, p).

### Physiological responses of rapeseed plants to salinity

**Fig. 3:**
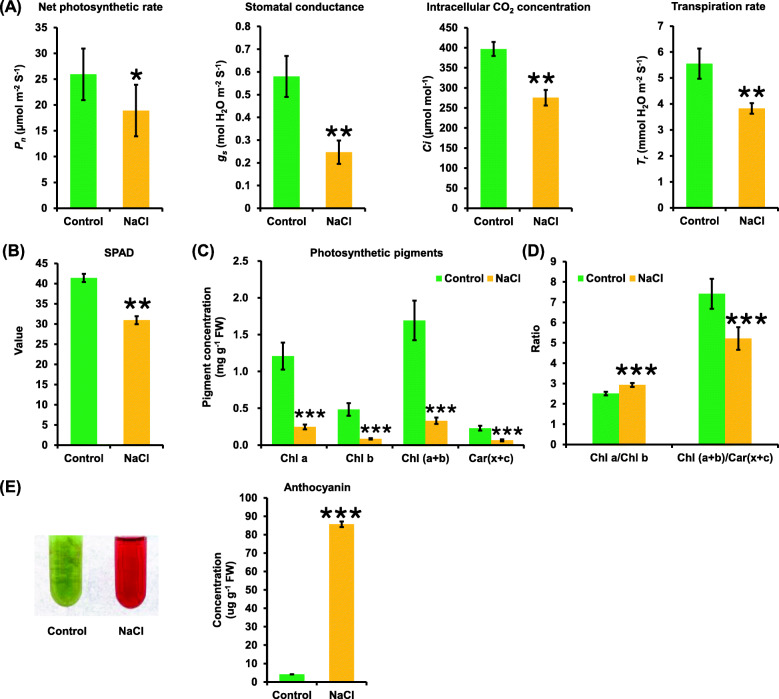
Photosynthetic characteristics and anthocyanin concentrations in rapeseed plants under the control and salt conditions. **a** Effect of salinity on net photosynthetic rate (*P*_*n*_, μmol m^− 2^ s^− 1^), stomatal conductance (*g*_*s*_, mol H_2_O m^− 2^ s^− 1^), intercellular CO_2_ concentration (*C*_*i*_, μmol mol^− 1^), and transpiration rate (*T*_*r*_, mmol H_2_O m^− 2^ s^− 1^) of the rapeseed plants under control and 200 mM NaCl conditions. **b**-**d** The SPAD values (**b**), photosynthetic pigment concentrations (**c**, **d**) and anthocyanin concentrations (**e**) in rapeseed plants under control and 200 mM NaCl conditions. Uniform rapeseed plants after 7-day seed germination were hydroponically grown under NaCl-free (control) for 10 days, and then they were transferred to the solution containing 200 mM NaCl for 5 days. Data are means (±SD), n = 5. Significant differences were determined using Student’s *t*-test: ns, not significant; *, *P* < 0.05; **, *P* < 0.01; ***, *P* < 0.001

Based on the differential morphologic analysis between the control and salinity conditions, the salinity-induced physiologic changes were further explored in rapeseed plants. The results showed a negative effect of salinity on the net photosynthesis rate, which was reduced by more than 20% (Fig. 3a). Besides the reduction in stoma number and conductance identified by scanning microscopy under salinity (Fig. 2a, d), stomatal conductance was reduced by more than a half (Fig. 3a). The decreased stomatal conductance was related to the reduction in the intracellular CO_2_ concentrations, which was approximately reduced by a quarter (Fig. 3a). In addition, the transpiration rate of the rapeseed plants was obviously lower under salinity than under the control condition (Fig. 3a).

The assay of SPAD values, representing leaf chlorophyll contents, also showed that salinity inhibited the photosynthesis of rapeseed plants (Fig. 3b). Further, the effect of salinity on photosynthetic pigments was investigated. Without exception, the concentrations of both total chlorophylls (including chlorophyll a and b) and carotenoids (including xanthophyll and carotene) were significantly reduced (Fig. 3c). The concentration ratio of chlorophyll *a* to chlorophyll *b* was obviously higher under salinity than that under the control condition (Fig. 3d). In addition, salinity reduced the ratio of total chlorophyll concentration to carotenoid concentration (Fig. 3d). Overaccumulated anthocyanin was also observed, which was validated by its significantly increased concentrations under salinity (Fig. 3e).

**Fig. 4:**
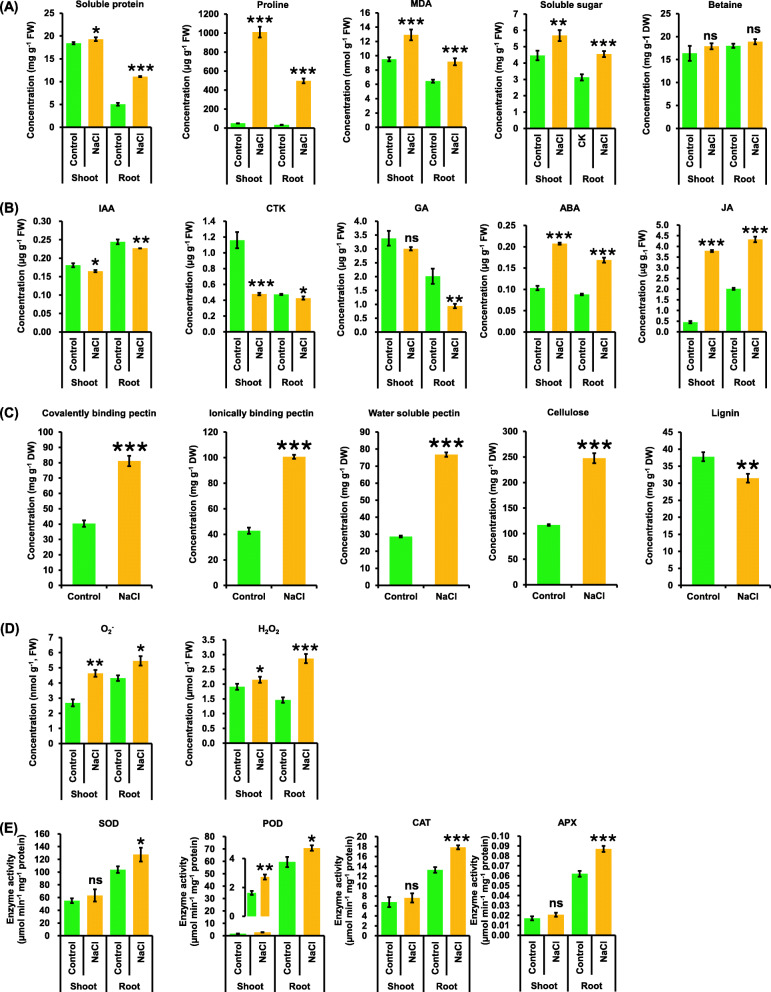
Some key metabolite profiling of rapeseed plants under the control and salt conditions. **a**-**e** Concentrations of some osmoregulation substances (A, including soluble protein, proline, MDA, soluble sugar and betaine), phytohormones (**B**, including IAA, CTK, GA, ABA, and JA), cell wall components (**c**, including covalently/ionically binding and water soluble pectin, cellulose, and lignin), reactive oxygen species (**d**, including O_2_^−^ and H_2_O_2_) and the activity of antioxidant enzymes (**e**, SOD, POD, CAT, and APX) in the shoots and roots of the rapeseed plants under control and 200 mM NaCl conditions. Uniform rapeseed plants after 7-day seed germination were hydroponically grown under NaCl-free (control) for 10 days, and then they were transferred to the solution containing 200 mM NaCl for 5 days. Data are means (±SD), *n* = 3. ns, not significant; *, *P* < 0.05; **, *P* < 0.01; ***, *P* < 0.001

Some key metabolites that might be involved in the SSR regulation were tested to further understand the physiological responses of rapeseed plants to salinity. The concentrations of soluble proteins, proline, malondialdehyde (MDA), and soluble sugar were significantly increased in the shoots and roots of rapeseed plants after exposure to salinity (Fig. 4a). In this study, no significant changes in the betaine concentrations were observed in both shoots and roots of the rapeseed plants under salinity compared with the control condition (Fig. 4a). Subsequently, phytohormone responses of rapeseed plants under salinity were investigated. In general, the concentrations of indoleacetic acid (IAA), cytokinin (CTK), and gibberellic acid (GA) were significantly lower under salinity than under the control condition in the rapeseed plants (Fig. 4b). However, the GA concentrations in the shoots showed no significant differences between the control and salinity conditions (Fig. 4b). On the contrary, the concentrations of abscisic acid (ABA) and jasmonic acid (JA) in the shoots and roots were significantly higher under salinity compared with the control condition (Fig. 4b).

Cell wall components were measured because the curl and changes in the cell wall ultrastructure were observed in the rapeseed leaves under salinity. Responses of covalently/ionically binding and water-soluble pectin, cellulose, and lignin to salinity are plotted in Fig. 4c. Compared with the control condition, the concentrations of covalent/ionic binding and water-soluble pectin, and cellulose in the shoots were about two-folds of those under the control condition (Fig. 4c). However, the lignin concentration was significantly decreased under salinity (Fig. 4c). The disorder of cell wall structure usually induces electrolyte leakage, which was often accompanied by ROS accumulation [26]. The superoxide anion (O_2_^−^) and hydrogen peroxide (H_2_O_2_) concentrations in the shoots and roots were significantly higher under salinity than that under the control condition (Fig. 4d). The ROS homeostasis is governed by a complicated regulation in ROS production and scavenging. The result showed that more ROS were accumulated under salinity than under the control condition (Fig. 4d). In response to salinity, the activities of superoxide dismutase (SOD), peroxidase (POD), catalase (CAT), and ascorbate peroxidase (APX) were significantly increased in the plant roots. However, the activities of SOD, CAT, and APX were not significantly altered in the shoots except that the POD activity was obviously elevated (Fig. 4e).

### Ionomic responses of rapeseed plants to salinity

**Fig. 5:**
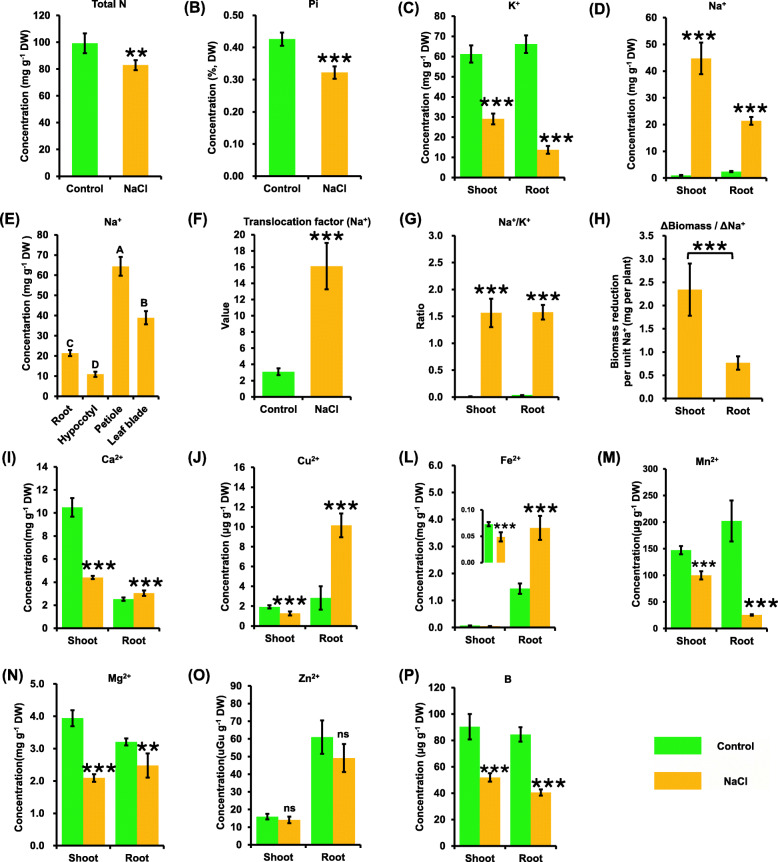
Ionomic profiling of rapeseed plants under the control and salt conditions. Uniform rapeseed plants after 7-day seed germination were hydroponically grown under NaCl-free (control) for 10 days, and then they were transferred to the solution containing 200 mM NaCl for 5 days. N, nitrogen; Pi, phosphate; K, potassium; Na, sodium; Ca, calcium; Mg, magnesium; Fe, iron; Mn, manganese; Cu, copper; Zn, zinc; B, boron. ns, not significant. Translocation factor (Na^+^) = [Na^+^ content]_shoot_/[Na^+^ content]_root_. Data are means (±SD), *n* = 3. ns, not significant; *, *P* < 0.05; **, *P* < 0.01; ***, *P* < 0.001

Subsequently, ICP-MS was used to assay the ionomic profiling between the control and salinity conditions. The results showed that most of the concentrations of mineral elements, including some macronutrients (N, P, K, Ca, and Mg) and micronutrients (Fe, Cu, Mn, Zn, and B), in the shoots and roots were obviously deceased under salinity compared with the control condition (Fig. 5a-p). Quantitative analysis by ICP-MS showed that Na^+^ concentrations were significantly increased in both shoots and roots under salinity (Fig. 5d). Moreover, the Na^+^ concentration in the shoots was higher than that in the roots (Fig. 5d). The Na^+^ concentration in the roots, hypocotyls, petioles, and leaf blades was measured in this study to further characterize the distribution of Na^+^ into plants. The analysis showed that the Na^+^ concentration was the highest in the petioles, followed by that in the leaf blades and in the roots, and the lowest in hypocotyls (Fig. 5e). The translocation factor of Na^+^ was significantly increased under salinity than that under the control condition (Fig. 5f). Under salinity, the Na^+^/K^+^ ratio was remarkably increased in both the shoots and roots (Fig. 5g). Biomass comparison analysis showed that the accumulation of Na^+^ resulted in a higher decrease in the biomass of shoots than that of roots (Fig. 5h).

Subsequently, the concentrations of some other metal cations, including Ca^2+^, Fe^2+^, Cu^2+^, Mg^2+^, Mn^2+^, and Zn^2+^, and a metalloid nutrient, namely B, were examined (Fig. 5i-p). In general, the Ca^2+^, Fe^2+^, and Cu^2+^ showed a similar response pattern under salinity. In detail, the concentrations of Ca^2+^, Fe^2+^, and Cu^2+^ in the shoots were significantly decreased; on the contrary, their concentrations were obviously increased in the roots (Fig. 5i-l). The Mg^2+^ and Mn^2+^ concentrations in both the shoots and roots were significantly decreased under salinity (Fig. 5m and n). Different from the above-mentioned cations, the Zn^2+^ concentrations in both the shoots and roots did not significantly change under salinity compared with the control condition (Fig. 5o). Also, the B concentrations in the shoots and roots were significantly lower under salinity than those under the control condition (Fig. 5p).

### Genome-wide transcriptional responses of rapeseed plants to salinity

After removing adapter sequences and low-quality reads, approximately 5.4 × 10^7^ clean reads of each sample were obtained. Total length of clean reads of 12 samples reached about 6.5 × 10^8^ nt with Q_20_ > 98% and Q_30_ > 95% (Supplementary Table S1). Most of *Pearson* correlation coefficients between each pair of biological replicates under the same treatment were higher than 0.90 (Supplementary Fig. S1), indicating that the transcriptome data were highly credible.

**Fig. 6:**
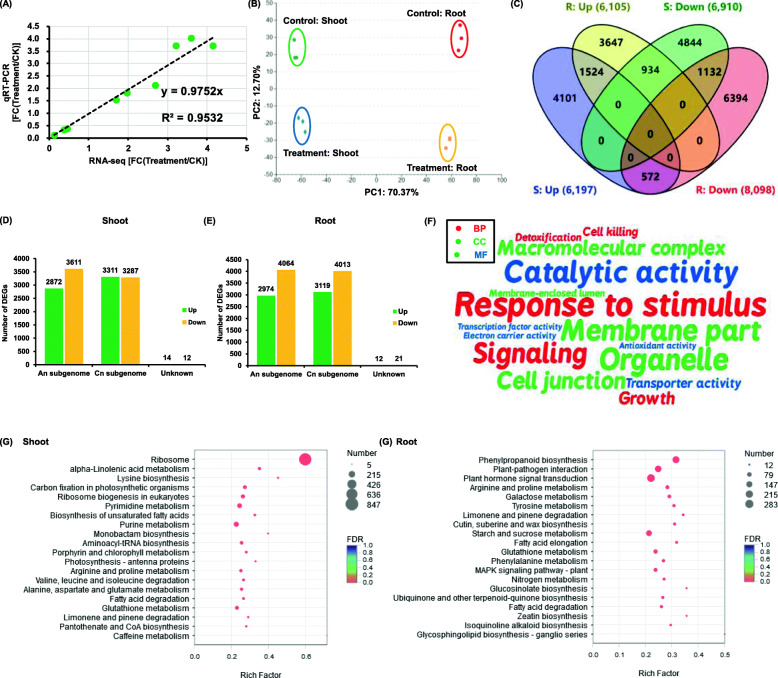
Overview of transcriptome sequencing data of rapeseed plants under the control and salt conditions. **a** Correlation analysis between the RT-qPCR assays and RNA-seq results. **b**, **c** Principal component analysis (B) and Venn diagram analysis (**c**) of the differentially expressed genes (DEGs) in the shoots (S) and roots (R) between the control and salt (treatment) conditions. The DEG numbers are listed in the brackets. **d**-**e** Volcano diagrams showing the DEGs between the control and salt (treatment) conditions in the shoots (**d**) and roots (**e**). Up, upregulation; down, downregulation. **f**-**g** GO (**f**) and KEGG pathway (**g**) enrichment analysis of global DEGs in the shoots and roots between the control and salt (treatment) conditions. For **f**, BP, biological process; CC, cellular component; MF, molecular function. Over-presentation of the GO items are delineated by the WordArt program. The bigger the font size, the more the corresponding GO categories. For **g**, the circle size indicates the number of DEGs, and the rich factor indicates the degree of enrichment of KEGG pathways involving the DEGs. For the transcriptome sequencing, uniform rapeseed plants after 7-day seed germination were cultivated under NaCl-free (control) for 10 days, and then they were changed to the solution containing 200 mM NaCl for 12 h

Subsequently, global differential gene expression of rapeseed was detected under 200 mM NaCl condition compared with the salt-free condition. First, 10 DEGs were selected to compare their expression consistency between the RT-qPCR assays and transcriptome sequencing. The results showed that most of the gene expression was highly correlated (*r* > 0.95) between the two assays (Fig. 6a). The principal component analysis revealed significant differences in the expression patterns between different treatments and different rapeseed tissues (Fig. 6b). A total of 13,107 and 14,203 genes were identified to be differentially expressed in the shoots and roots under salinity, respectively (Fig. 6c). The number of downregulated DEGs in both the shoots and roots was larger than that of upregulated DEGs (Fig. 6c). Further, the effect of salinity on the homeolog expression bias was surveyed between the An and Cn subgenomes of allotetraploid rapeseed (Fig. 6d, e). In general, the DEGs on the Cn subgenomes were more abundant than those on the An subgenome in both shoots and roots (Fig. 6d, e).

Before in-depth investigation of the specific responses of DEGs, molecular function (MF) of the DEGs showing the largest changes in the expression levels under salinity was explored. As shown in Table 1, the DEGs of late embryogenesis abundant (LEA) proteins, dehydrin, sugar and N transporters, defensins, and other stress-related proteins showed very high expression changes under salinity. Further, the enrichment analysis of GO terms, including MF, cellular component (CC), and biological process (BP), was performed to characterize main biological roles of the DEGs under salinity. In spite of the shoots or the roots under salinity, the most highly enriched GO term for BP was the response to stimuli, signaling, growth, and cell killing (Fig. 6f). However, the membrane part, organelle, and cell junction were the three most enriched items in the CC category (Fig. 6f). In the MF annotation, the catalytic and transporter activities were the two most enriched items (Fig. 6f). The KEGG database was used to further identify the active pathways involving the responses of *B. napus* to salinity. In the shoots, the pathways for pyrimidine and purine metabolism, porphyrin and chlorophyll metabolism, and carbon fixation in photosynthetic organisms were highly enriched (Fig. 6g). In the roots, a large proportion of DEGs were mainly involved in the phenylpropanoid biosynthesis, plant hormone signal transduction, wax biosynthesis, and carbon (including starch and sugar)-nitrogen metabolism (Fig. 6g).

**Table 1:** Twenty differentially expressed genes with the highest expression changes under salinity in the shoots and roots

	Gene ID	Fold change	Regulation	Annotation
Shoots	BnaA02g07120D	26,749	Up	Non-specific lipid-transfer protein 4
	BnaA02g29990D	14,762	Up	Nitrile-specifier protein 5-like
	BnaC09g48810D	12,166	Up	Late embryogenesis abundant protein 46
	BnaA10g20120D	5989	Up	Bidirectional sugar transporter SWEET15-like
	BnaC02g45160D	4650	Up	Dehydrin Rab18-like
	BnaA03g13140D	4020	Up	Non-specific lipid-transfer protein 3
	BnaC01g01500D	3753	Up	Cytochrome P450 81F1-like
	BnaA10g24180D	3753	Up	Late embryogenesis abundant protein 46
	BnaC09g43920D	3441	Up	Bidirectional sugar transporter SWEET15-like
	BnaC03g32950D	3316	Up	Stress-induced protein KIN2
	BnaA05g30910D	0.0005	Down	Response to water deprivation
	BnaC04g33320D	0.0015	Down	Pentatricopeptide repeat-containing protein
	BnaC03g01280D	0.0015	Down	Purine-uracil permease NCS1
	BnaC01g22240D	0.0017	Down	N-lysine methyltransferase
	BnaA01g18330D	0.0022	Down	N-lysine methyltransferase
	BnaA03g38630D	0.0025	Down	Pathogenesis-related protein 1-like
	BnaA02g15510D	0.0029	Down	Endoglucanase 9-like
	BnaC02g20750D	0.0031	Down	Endoglucanase 9-like
	BnaC02g23620D	0.0034	Down	Defensin-like protein 3
	BnaC08g43380D	0.0039	Down	High-affinity nitrate transporter 2.1
Roots	BnaC03g32950D	3947	Up	Stress-induced protein KIN2
	BnaA10g24180D	3304	Up	Late embryogenesis abundant protein 46
	BnaC06g22120D	2469	Up	Defensin-like protein 3
	BnaCnng27850D	1362	Up	Dehydrin Rab18
	BnaA09g05190D	1283	Up	Bidirectional sugar transporter SWEET12
	BnaC05g41300D	1115	Up	Universal stress protein PHOS34-like
	BnaCnng67820D	1036	Up	Sulfate transporter 3.1
	BnaC09g48810D	939	Up	Late embryogenesis abundant protein 46
	BnaC06g21150D	937	Up	Alcohol dehydrogenase class-P
	BnaA09g49440D	864	Up	Protein phosphatase 2C
	BnaA02g20580D	0.0004	Down	GDSL esterase/lipase
	BnaC09g42600D	0.0005	Down	Senescence-associated carboxylesterase 101
	BnaA05g34160D	0.0005	Down	UDP-glycosyltransferase 83A1
	BnaA09g51570D	0.0007	Down	Cytochrome P450 78A6
	BnaC04g19370D	0.0007	Down	S-type anion channel SLAH1
	BnaC07g34090D	0.0007	Down	Protease
	BnaA04g03090D	0.0007	Down	Glucan endo-1,3-beta-glucosidase
	BnaC01g02200D	0.0007	Down	Protein RADIALIS-like 3
	BnaA03g35580D	0.0007	Down	Cytochrome P450 705A22-like
	BnaC03g29930D	0.0008	Down	Cysteine-rich receptor-like protein kinase 37

Note: *down* downregulation; *up* upregulation

### Transcriptional responses of photosynthesis and anthocyanin biosynthesis-related genes to salinity

**Fig. 7:**
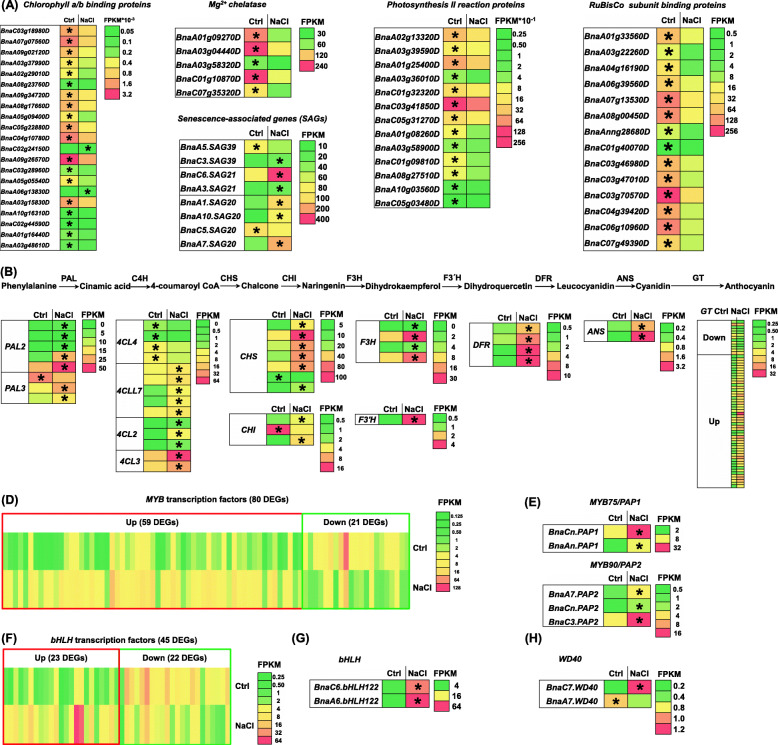
Differential expression profiling of the genes involved in photosynthesis and anthocyanin biosynthesis in the shoots between the control (Ctrl) and salt conditions. **a** Differential expression profiling of the genes involved in photosynthesis. **b** The anthocyanin biosynthesis pathway in plants and differential expression profiling of the genes involved in anthocyanin biosynthesis. ANS, anthocyanidin synthase; C4H, cinnamic acid 4-hydroxylase; CHI, chalcone-flavanone isomerase; CHS, chalcone synthase; 4CL, coumaroyl-CoA synthase; DRF, dihydroflavonol 4-reductase; F3H, flavanone 3-hydroxylase; F3’H, flavanone 3′-hydroxylase; GT, glycosyltransferase; PAL, phenylalanine ammonia lyase. **c** General expression profiling and number of the differentially expressed *MYB* transcription factor genes. **d** Differential expression profiling of the *PAP1* (*MYB75*) and *PAP2* (*MYB90*). PAP, production of anthocyanin pigment. **e** General expression profiling and number of the differentially expressed *bHLH* transcription factor genes. **f** Differential expression profiling of the *bHLH122* family genes. **g** Differential expression profiling of the *WD40* family genes. For the transcriptome sequencing, uniform rapeseed plants after 7-day seed germination were hydroponically grown under NaCl-free (control) for 10 days, and then they were transferred to the solution containing 200 mM NaCl for 12 h. Up, upregulation; down, down regulation. The heatmaps show gene expression levels as indicated by the FPKM values. The differentially expressed genes presenting higher expression levels between the control (Ctrl) and salt (200 mM NaCl) conditions are denoted by asterisks

Figure 3a-c shows the serious degradation of chlorophyll and the inhibition of photosynthesis in the rapeseed plants exposed to salinity. In the transcriptomic analysis, we found that chlorophyll biosynthesis-related genes, *Mg*^*2+*^ *chelatase*, *senescence-associated genes (SAGs)*, and genes encoding photosynthesis II reaction proteins and RuBisCo subunit binding proteins were significantly down-regulated under salinity (Fig. 7a).

Anthocyanins, functioning as a kind of non-enzyme antioxidant against the stresses, are produced mainly in a phenylpropanoid-dependent manner. In the process of anthocyanin biosynthesis, first, phenylalanine is converted into cinnamic acid by phenylalanine ammonia lyase (PAL), and disintegrates into several pathways at coumaroyl CoA. Subsequently, coumaroyl CoA-deriving flavonoid is formed by the catalysis of chalcone synthase (CHS), which then triggers the biosynthesis of flavonol, cyanidin, and anthocyanin (Fig. 7b). In Fig. 3e, anthocyanins were found to be over-accumulated in the leaves of rapeseed plants after exposure to salinity. Further, transcriptional profiles of the genes involved in the anthocyanin biosynthesis were investigated under salinity. The results showed that about 95% of the DEGs were significantly up-regulated under salinity (Fig. 7b). The MYB-bHLH-WDR (MBW) complex is critical for the anthocyanin biosynthesis [27]. A major proportion (75%) of the genome-wide DEGs of *BnaMYBs* was found to be induced by salinity (Fig. 7c). The transcript levels of *BnaA7.PAP1* and *BnaA7.PAP2* were remarkably higher under salinity than under the control condition (Fig. 7d). Half (50%) of the genome-wide DEGs of *BnabHLHs* were found to be induced by salinity (Fig. 7e). The two *bHLH122* homologs (*BnaA6.bHLH122* and *BnaC6.bHLH122*), significantly induced by salinity, showed the highest expression abundances (Fig. 7e, f). Only the differential expression of *BnaA7.WD40* and *BnaC7.WD40* was identified (Fig. 7g) in terms of the genome-wide *WD40* genes.

### Transcriptional responses of genes involved in proline and cell wall biosynthesis, and ROS production and scavenging to salinity

**Fig. 8:**
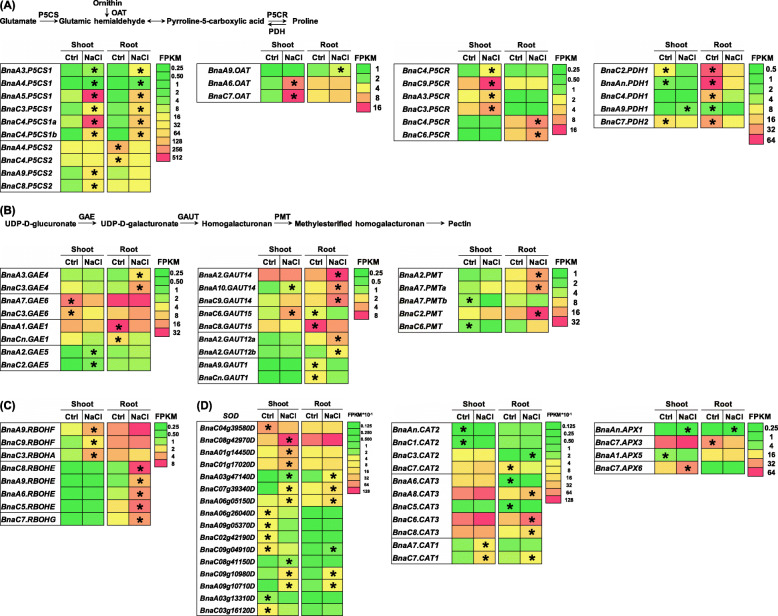
Differential expression profiling of genes involved in proline and pectin biosynthesis, reactive oxygen species (ROS) production and scavenging in rapeseed plants between the control (Ctrl) and salinity conditions. **a** Proline biosynthesis pathway and differential expression profiling of the involving genes. OAT, ornithine aminotransferase; P5CR, pyrroline-5-carboxylate reductase; P5CS, pyrroline-5-carboxylate synthase; PDH, proline dehydrogenase. **b** Pectin biosynthesis pathway and differential expression profiling of the involving genes. GAE, UDP-glucuronate 4-epimerase; GAUT, galacturonosyl transferase; PMT, pectin methyltransferase. **c** Differential expression profiling of the genes involved in ROS production. RBOH, respiratory burst oxidase homologs. **d** General expression profiling of differentially expressed antioxidant enzyme genes. APX, ascorbate peroxidase; CAT, catalase; SOD, superoxide dismutase. For the transcriptome sequencing, uniform rapeseed plants after 7-day seed germination were cultivated under NaCl-free (control) for 10 days, and then they were transferred to the solution containing 200 mM NaCl for 12 h until sampling. The heatmaps show gene expression levels as indicated by the FPKMvalues. The differentially expressed genes presenting higher expression levels between the control (Ctrl) and salt (200 mM NaCl) conditions are denoted by asterisks

Proline is a main osmotic adjustment substance to maintain cellular homeostasis under environmental stress [28]. Proline biosynthesis begins with the conversion of glutamate into glutamic semialdehyde by pyrroline-5-carboxylate synthase (P5CS) or the conversion of ornithine into glutamic semialdehyde by ornithine aminotransferase (OAT), which is then converted into pyrroline-5-carboxylate reductase (P5CR), and, finally, into proline through the catalysis of OAT (Fig. 8a). Under salinity, proline was found to be over-accumulated; the expression of *P5CS*, *OAT*, and *P5CR* was significantly upregulated in both shoots and roots (Fig. 8a). On the contrary, the expression of *BnaPDHs* was significantly decreased (Fig. 8a). Among the DEGs that were involved in the proline biosynthesis, *BnaA5.P5CS1*, *BnaC4.P5CS1a*, *BnaC7.OAT*, *BnaC9.P5CR*, and *BnaAn.PDH1*, showing higher expression levels and fold changes (Fig. 8a), might be mainly responsible for the salinity-induced proline production.

In the morpho-physiologic analysis, significant alterations were found in the cell wall ultrastructure and components (Figs. 2, 4c). This study focused on pectin among the cell wall components. Pectin biosynthesis begins with UDP-glucuronates, which were then erased by UDP-glucuronates 4-epimerase, galacturonic transferase, and pectin methyltransferase (PMT), and, eventually, converted into pectin (Fig. 8b). The RNA-seq results showed that a larger proportion of the pectin biosynthesis-related genes were induced by salinity in the shoots or roots (Fig. 8b). Among these upregulated genes, *BnaA2.GAUT14* and *BnaC2.PMT*, showing higher expression levels and fold changes (Fig. 8b), might play core roles in the pectin biosynthesis under salinity.

R*espiratory burst oxidase homolog* (*RBOH*) genes encoding NADPH oxidases are pivotal for the ROS production [29]. All the eight *RBOH* DEGs were significantly upregulated in the shoots or roots under salinity (Fig. 8c). In response to salinity, a larger proportion of the genes encoding SOD, CAT, and APX was upregulated in the shoots or roots (Fig. 8d). Among the antioxidant enzyme genes, *BnaC8.SOD*, *BnaC6.CAT3*, and *BnaC7.APX6*, showing higher expression levels and fold changes (Fig. 8d), might be essential for the ROS scavenging under salinity.

### Transcriptional responses of phytohormone biosynthesis-related genes to salinity

The concentrations of IAA, CTK, and GA were markedly decreased and the concentrations of ABA and JA were obviously increased under salinity (Fig. 4b). Hence, the present study investigated transcriptional responses of the phytohormone metabolism-related genes to salinity in rapeseed.

**Fig. 9:**
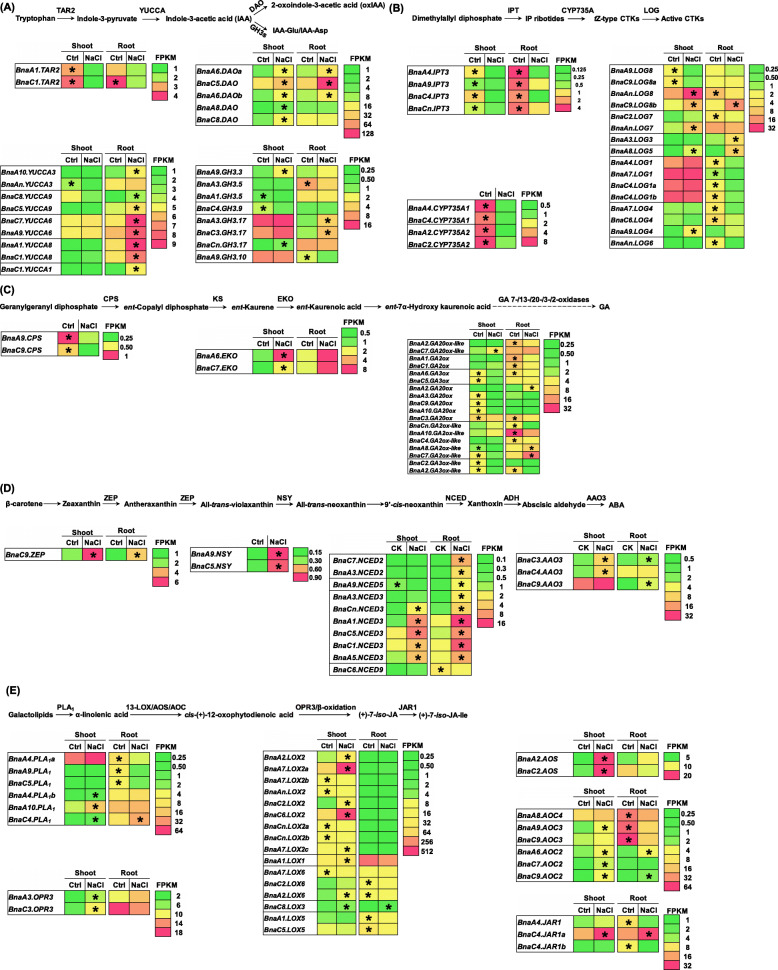
Differential expression profiling of genes involved in phytohormone biosynthesis in the rapeseed plants between the control (Ctrl) and salt conditions. **a**-**e** Biosynthesis pathways and differential expression profiling of the genes involved in auxin (IAA, **a**), cytokinin (CTK, **b**), gibberellin (GA, **c**), abscisic acid (ABA, **d**), and jasmonic acid (JA, E). AAO, abscisic aldehyde oxidase; ADH, alcohol dehydrogenase; AOC, allene oxide cyclase; AOS, allene oxide synthase; CPS, *ent*-copalyl diphosphate SYNTHETASE; DAO, Dioxygenase for Auxin Oxidation; EKO, *ent*-kaurene 19-oxidase; GAox, GA oxidase; GH3s, Gretchen Hagen 3 s; KS, *ent*-kaurene synthase; LOX, lipoxygenase; NCED, 9-*cis*-epoxycarotenoid dioxygenase; OPR, 12-oxo-phytodienoic acid reductase; PLA_1_, phospholipase A_1_; ZEP, zeaxanthin epoxidase; ZSY, neoxanthin synthase. For the transcriptome sequencing, uniform rapeseed plants after 7-day seed germination were cultivated under NaCl-free (control) for 10 days, and then they were transferred to the solution containing 200 mM NaCl for 12 h. The heatmaps show gene expression levels as indicated by the FPKM values. The differentially expressed genes presenting higher expression levels between the control and 200 mM NaCl conditions are denoted by asterisks

Auxin biosynthesis begins with the conversion of tryptophan into indole-3-pyruvate, and finally into IAA, regulated by *TAR2* and *YUCCA* (Fig. 9a). The principal auxin degradation pathways in *Arabidopsis* include oxidation by *dioxygenase for auxin oxidation* and conjugation by *Gretchen Hagen 3 s* [30]. In general, the expression of *BnaTAR2* was significantly downregulated under salinity, whereas the expression of *BnaYUCCA* was significantly up-regulated in the shoots and roots (Fig. 9a). Under salinity, most of the *BnaDAO* and *BnaGH3* DEGs were significantly up-regulated in the shoots and roots (Fig. 9a). CTK biosynthesis begins with dimethylallyl diphosphate that finally is converted into active CTKs by IPT, CYP735A, and LOG. Under salinity, the expression levels of all the *BnaIPT3* and *BnaCYP735A1s* and most *BnaLOG* DEGs were significantly down-regulated (Fig. 9b). GA biosynthesis begins with geranylgeranyl diphosphate that was converted into GA by CPS, KS, EKO, and GA 7−/13−/20−/3−/2-oxidases. Under salinity, the expression levels of *BnaCPSs* were significantly decreased, whereas the *BnaEKO*s were significantly up-regulated (Fig. 9c).

ABA biosynthesis begins with the conversion of β-carotene into ABA by a series of enzymes, such as ZEPs, NSYs, NCEDs, and AAOs (Fig. 9d). Under salinity, the expression of *BnaZEPs*, *BnaNSYs*, *BnaNCED2s*, and *BnaAAO3s* were significantly up-regulated (Fig. 9d). The *BnaC9.ZEP*, *BnaA9.NSY*, and *BnaA1.NCED3*, showing higher expression levels and fold changes (Fig. 9d), might play dominant roles in the ABA biosynthesis. JA biosynthesis begins with the conversion of galactolipids into JA by a series of enzyme genes, include PLA_1_, LOX, AOS, AOC, OPR, and JAR1 (Fig. 9e). The JA biosynthesis-related genes showed a distinct transcriptional response to salinity. The DEGs of both *BnaOPR3s* and *BnaAOSs* were significantly up-regulated in the rapeseed plants (Fig. 9e). However, other JA biosynthesis-related genes DEGs did not show consistent responses to salinity (Fig. 9e), implying a complex regulatory network of the JA biosynthesis under salinity. Among the DEGs, *BnaA7.LOX2a*, *BnaC6.LOX2*, *BnaA2.AOS*, *BnaC2.AOS*, and *BnaC4.JAR1a* showed enhanced expression levels and fold changes (Fig. 9e), and might play dominant roles in the JA biosynthesis.

### Transcriptional responses of Na^+^/K^+^ transporter genes to salinity

**Fig. 10:**
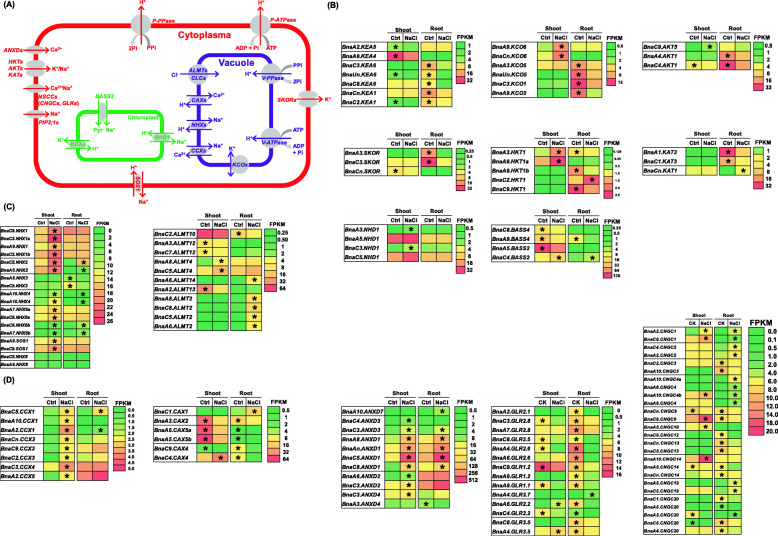
Differential expression profiling of genes involved in the transport of sodium (Na^+^), chlorion (Cl^−^), potassium (K^+^), and calcium (Ca^2+^) ions in rapeseed plants between the control (Ctrl) and salt conditions. **a** A molecular model showing the genes involved in the transport of Na^+^, K^+^, and Ca^2+^ in plants. **b-d** Differential expression profiling of the genes involved in the transport of K^+^ (**b**), Na^+^/Cl^−^ (**c**), and Ca^2+^ (**d**) in the rapeseed plants between the control and 200 mM NaCl conditions. ALMT, aluminum-activated malate transporter; AKT/KAT, Arabidopsis K^+^ transporter; ANXD, annexin D; BASS, bile acid sodium symporter; CAX, cation exchanger; CCX, cation calcium exchanger; CNGC, cyclic nucleotide-gated channel; GLR, glutamate-like receptor; HKT, high-affinity K^+^ transporter type; KEA, K^+^ efflux antiporter; KCO, two-pore K^+^ channel; NHX/NHD, Na^+^/H^+^ antiporter; SKOR, stelar K^+^ outward rectifier; SOS, salt overly sensitive. For the transcriptome sequencing, uniform rapeseed plants after 7-d seed germination were hydroponically grown under NaCl-free (control) for 10 d, and then they were transferred to the solution containing 200 mM NaCl for 12 h. The heatmaps show gene expression levels as indicated by the FPKM values. The differentially expressed genes presenting higher expression levels between the control and 200 mM NaCl conditions are denoted by asterisks

Among the numerous DEGs, much attention was paid to the genes implicated in nutrient ion homeostasis, which was crucial for the resistance of rapeseed plants to salinity. A molecular model showing the genes responsible for the transport of Na^+^, K^+^, and other cations was plotted in Fig. 10a. The transcriptomics results showed that most of the K^+^ transporter genes, including the chloroplast-localized K^+^ efflux transporter gene *KEA* (*K*^*+*^ *efflux antiporter*), the vacuolar K^+^ influx transporter gene *KCO* (*two-pore K*^*+*^ *channel*), the plasma membrane-localized K^+^ influx transporter genes *AKT/KAT* (*Arabidopsis K*^*+*^ *transporter*) and *HKT* (*high-affinity K*^*+*^ *transporter type*), and the K^+^ efflux gene *SKOR* (*stelar K*^*+*^ *outward rectifier*), were downregulated under salinity (Fig. 10b). The *Na*^*+*^*/H*^*+*^ *antiporter* (*NHX*) genes, particularly the tonoplast-localized *NHX1* and *NHX2* responsible for vacuolar Na^+^ compartmentation and the plasma membrane-localized *SOS1*/*NHX7* regulating cell Na^+^ extrusion, were upregulated (Fig. 10c). The downregulation of the chloroplast-localized *bile acid sodium symporter* (*BASS*) regulating Na^+^ influx, particularly *BnaA5.BASS2*, and the upregulation of the *NHD* genes regulating Na^+^ efflux, particularly *BnaC3.NHD1* (Fig. 10c) might contribute to mitigating the chloroplast damages induced by excess Na^+^. Besides, excess NaCl also induced the expression of most of the *ALMT* (*aluminum-activated malate transporter*) genes involved in vacuolar chloride (Cl^−^) sequestration (Fig. 10c).

Subsequently, the expression of some other cation transporter genes, including *cation calcium exchanger* (*CCX*), *cation exchanger* (*CAX*), *annexin D* (*ANXD*), *glutamate-like receptor* (*GLR*), and *cyclic nucleotide-gated channel* (*CNGC*) was investigated, which might be involved in the Na^+^ homeostasis under salinity. Under salinity, the increased expression of *BnaCCXs*, the reduced expression of *BnaCAXs*, and the enhanced expression of *BnaANXDs* might contribute to diminishing the cytosolic Na^+^ and enhancing cytosolic Ca^2+^ concentrations (Fig. 10d). Most of the expression of the plasma membrane-localized *non-selective cation channels* (*NSCCs*), including *GLRs* and *CNGCs*, was down-regulated (Fig. 10d), which might prevent excess Na^+^ from entering the cytoplasm.

### Transcriptional response of other transporter genes to salinity

**Fig. 11:**
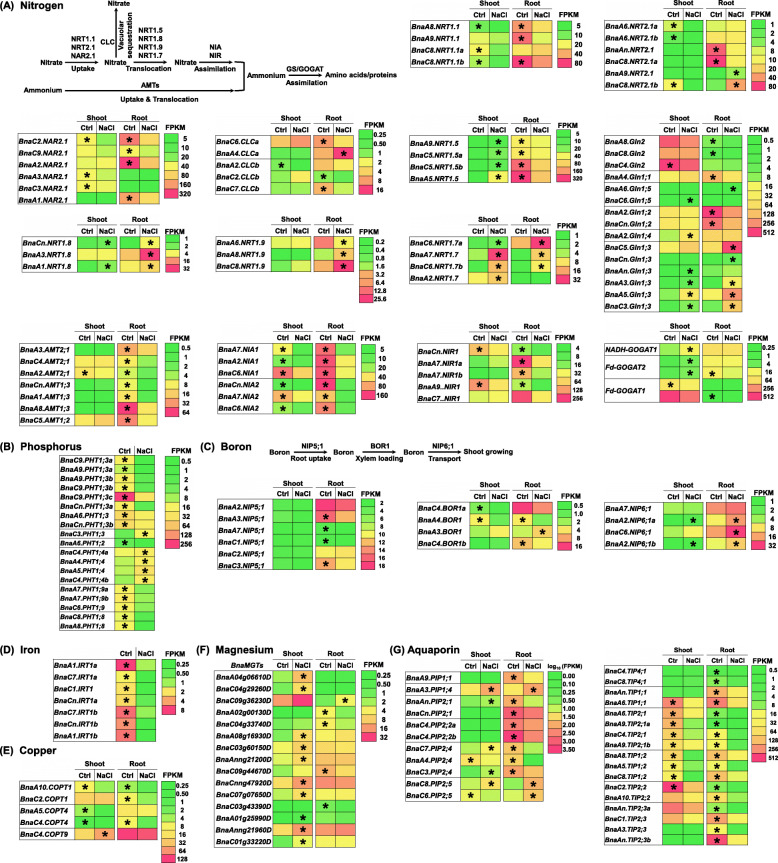
Differential expression profiling of genes involved in the transport of nitrogen (N), phosphorus (P), boron (B), iron (Fe), copper (Cu), magnesium (Mg), and the aquaporin genes in the rapeseed plants between the control (Ctrl) and salt conditions. Differential expression profiling of the genes involved in the transport of N (**a**), P(**b**), B (**c**), Fe (**d**), Cu (**e**), Mg (**f**), and the aquaporin genes (**g**) in the rapeseed plants between the control and 200 mM NaCl conditions. AMT, ammonium transporter; CLC, chloride channel; COPT, copper transporter; GS, glutamine synthetase; GOGAT, glutamine-2-oxoglutarate aminotransferase; IRT, iron transporter; NIA, nitrate reductase; NIR, nitrite reductase; NIP, nodulin 26-like intrinsic protein; NRT, nitrate transporter; MGT, magnesium transporter; PHT, phosphate transporter; PIP, plasma membrane intrinsic protein; TIP, tonoplast intrinsic protein. For the transcriptome sequencing, uniform rapeseed plants after 7-day seed germination were cultivated under NaCl-free (control) for 10 days, and then they were changed to the solution containing 200 mM NaCl for 12 h. The heatmaps show gene expression levels as indicated by the FPKM (values. The differentially expressed genes presenting higher expression levels between the control and 200 mM NaCl conditions are denoted by asterisks

Under salinity, N metabolism of plants was significantly changed [31]. A molecular model showed the genes involved in the N metabolism in plants (Fig. 11a). Under salinity, the nitrate transporter genes responsible for N uptake, including *NRT1.1*, *NRT2.1*, and *NAR2.1*, were significantly downregulated (Fig. 11a). Most of the vacuolar nitrate influx transporter genes, *chloride channels* (*CLCs*), also showed decreased expression levels under salinity than under the control condition. The root nitrate xylem loading genes, *BnaNRT1.5 s*, and root nitrate xylem unloading genes, *BnaNRT1.8 s*, were upregulated and downregulated under salinity, respectively, contributing to a higher retention of nitrate in the roots against the salinity. The nitrate transporter genes responsible for N recycling from senescent leaves to new organs, *NRT1.7* and *NRT1.9*, showed enhanced expression under salinity than under the control. In addition, the ammonium transporter genes, *AMTs*, were repressed by salinity. The *nitrate reductase* (*NIA1* and *NIA2*) and *nitrite reductase* (*NIR*) genes were downregulated, whereas the glutamine synthetase (GS) and *glutamine-2-oxoglutarate aminotransferase* (*GOGAT*) genes were induced by salinity.

Under salinity, most of phosphate (Pi) transporter genes were significantly down-regulated, especially *BnaC9.PHT1;3c* (Fig. 11b). Salinity obviously repressed the expression of B uptake channel genes, *BnaNIP5;1 s*, and B transporter genes, *BnaBOR1s* and induced the expression of *NIP6;1 s*, boric acid channels for preferential transport of boron to growing shoot tissues (Fig. 11c). The transporter genes (including *IRT1s*, *COPTs*, and *MGTs*) involved in the uptake and transport of Fe^2+^, Cu^2+^, and Mg^2+^ showed obvious downregulation in the roots under salinity (Fig. 11d-f). Under salinity, most of aquaporin genes, particularly *BnaC4.PIP2;2b* and *BnaC2.TIP2;2*, were significantly down-regulated in both the shoots and roots (Fig. 11f).

## Discussion

Salinity conditions severely inhibit plant growth, yield, and crop quality worldwide. Allotetraploid rapeseed (A_n_A_n_C_n_C_n_, 2*n* = 4*x* = 38), a major glycophytic oil crop, is highly susceptible to salinity. Understanding the physiological and molecular mechanisms underlying the rapeseed salinity resistance is a promising and cost-effective strategy for developing highly salt-resistant rapeseed cultivars.

### Transcriptomics-assisted dissection into morpho-physiologic responses of rapeseed plants to salinity

Different crops adapt to different ranges of salt concentrations, and different NaCl concentrations have different effects on plants [32–35]. NaCl concentrations of 0 mM, 50 mM, 100 mM,150 mM, 200 mM, and 250 mM were used to treat the rapeseed plants in this study so as to select the NaCl concentrations suitable for studying rapeseed SSR. The rapeseed plants did not show obvious growth inhibition at the NaCl concentrations of 50 mM, 100 mM, and 150 mM (Fig. 1a, b). However, the rapeseed seedlings showed excessive salt-induced plant damages with 250 mM NaCl, including early senescent leaves, inhibited roots, and, finally, plant death (Fig. 1a, b), which was not suitable for the crop SSR study. Finally, the NaCl concentration suitable for studying salinity of rapeseed was determined to be 200 mM, which was also widely used in other studies [35–39]. It might be concluded that 200 mM NaCl could be used as a universal condition for the crop SSR study under hydroponic culture.

Stomata, affected by environmental stimuli, is very important for the regulation of gas exchange between the leaf intracellular tissues and the external environment. A previous study shows that the stomatal valve also correlates with the net photosynthesis rate [40]. In this study, the salinity reduced the stoma number and conductance, and also resulted in the detached chloroplasts and chlorophyll degradation (Fig. 2), all of which might further lead to the photosynthesis inhibition (Fig. 3a–d). The transcriptomic data also showed that the photosynthesis-related KEGG pathway was highly accumulated (Fig. 6g), and the genes involved in the chlorophyll biosynthesis pathway were significantly downregulated under salinity (Fig. 7a). As a functional index of the photosynthetic pigments and light adaptation [41], the concentration ratio of chlorophyll *a* to chlorophyll *b* was obviously higher under salinity than under the control condition (Fig. 3d), indicating that salinity had a more obvious inhibitory effect on chlorophyll *b* than on chlorophyll *a*. In addition, the concentration ratio of total chlorophylls to carotenoids suggested the occurrence of senescence, stresses, or damages to photosynthesis [41], whereas salinity reduced the concentration ratio of total chlorophylls to carotenoids (Fig. 3d), indicating that chlorophylls were more sensitive to salt-induced damages.

Anthocyanins are important secondary metabolites and nonenzymatic antioxidants in plants [27]. Both the physiologic and transcriptomic data confirmed anthocyanin overaccumulation under salinity (Figs. 3e and b–g), which might protect the rapeseed leaves from excess photo-induced damages under salinity. Moreover, through a transcriptomic analysis, significant upregulation of some central genes that might be involved in the salinity-induced anthocyanin biosynthesis was identified, such as *BnaCn.PAP1*, *BnaC3.PAP2*, *BnaA6.bHLH122*, and *BnaC7.WD40* (Fig. 7c–g). Previous studies showed that the osmotic modulator levels significantly changed under salinity [31]. Under salinity, soluble protein, proline, MDA, and soluble sugar concentrations significantly increased (Fig. 4a). However, no significant alteration of betaine was found under salinity in this study, which was different from previous findings ([31, 42]. This result indicated that betaine might not be involved in the osmotic regulation of rapeseed SSR.

### Transcriptomics-assisted dissection into ionomic responses of rapeseed plants to salinity

Reducing Na^+^ content and increasing K^+^ content is key to improving plant salinity resistance. Under salinity, the Na^+^/K^+^ ratio significantly increased, affecting a variety of metabolic reactions in plants [43]. In this study, the Na^+^ concentration was found to be higher in the shoots than in the roots under salinity (Fig. 5d), which might further lead to a higher degree of biomass reduction in the shoots (Fig. 5h). The roots were first subjected to salinity, which had an enhanced inhibitory effect on the shoots than on the roots. The transcriptomic results showed that 13,107 and 14,203 DEGs were identified in the shoots and roots, respectively (Fig. 6c, d). The number of DEGs in the roots was obviously larger than that in the shoots, which might be attributed to the fact that the roots were first subjected to salinity.

In addition to reducing the cation concentration, the salinity condition caused an obvious decrease in the B concentration (Fig. 5), which was consistent with the downregulation of the B-uptake channel genes *BnaNIP5;1 s* (Fig. 11c). Under salinity, the rapeseed leaves became much thicker, more curved, and fragile (Fig. 1a), which was similar to the symptoms caused by B deficiency in rapeseed plants [44]. Salinity also had a significant effect on the cell wall ultrastructure and components (mainly pectin) (Fig. 4c), relative water content [45], and aquaporin activities (Fig. 11), which were regulated by the B nutrient status [44]. In rapeseed, B deficiency was shown to aggravate the plant growth inhibition caused by salinity [46]. It was concluded that a close crosstalk might exist between B and salinity, and suitable exogenous B application might alleviate the salinity-induced damages in plants.

### Transcriptomics-assisted identification of core members of salinity-responsive gene families in rapeseed

In this study, the morphological and physiological changes were identified in rapeseed plants under salinity condition (Figs. 1, 2, 3, 4 and 5). Further, through high-resolution transcriptomics profiling, the central salinity-responsive genes that were involved in the biosynthesis of osmoregulatory substances, cell wall components, antioxidant enzymes, phytohormones and the transport of mineral nutrients were identified and characterized (Figs. 7, 8, 9, 10 and 11). Proline, a key osmoregulatory substance in plant salinity resistance, may participate in the regulation of phytohormone metabolism under short-term of salt stress [47]. Under salinity condition, the characterized core genes that were implicated in the proline biosynthesis, including *BnaA5.P5CS1*, *BnaA7.OAT*, *BnaC9.P5CR*, and *BnaAn.PDH1* (Fig. 8a) would provide key genes for the molecular modulation of rapeseed salinity resistance. Under salt shock, three genes (*BnaC08g42970D*, *BnaC6.CAT3*, and *BnaC7.APX6*) encoding SOD, CAT, and APX (Fig. 8c), respectively were proposed to play core roles in the salinity-induced ROS scavenging, which contributed to relieving cell damages. Phytohormones, regulating plant development and modulates abiotic stress resistance [48], were significantly changed under salinity condition in this study (Fig. 9). NCED is a rate-limiting enzyme for the ABA biosynthesis, and *OsNCED5* overexpression, increasing ABA levels, enhances tolerance to rice salt resistance [48]. In addition, JA enhances the potato plant resistance to salt stress in vitro [49]. In this study, several key *NCED3* and *JAR* homologs, such as *BnaA1.NCED3* and *BnaC4.JAR1a*, were identified to be core genes regulating the ABA and JA biosynthesis, respectively (Fig. 9), which would provide key genes for fine tuning of phytohormone homeostasis under salinity conditions. Na^+^/K^+^ homeostasis is essential for salt stress adaptation. NHX-mediated compartmentation of Na^+^ into vacuoles is critical for plant salt stress resistance [50]. In this study, *BnaC2.NHX1a* and *BnaC9.SOS1*, identified as the core genes regulating vacuolar Na^+^ sequestration and Na^+^ efflux (Fig. 10) respectively, were proposed to elite gene resources for maintaining Na^+^ homeostasis. Taken together, transcriptomics-assisted identification and characterization of core members of salinity-responsive gene families will provide elite gene resources for genetic modification of rapeseed salinity stress resistance.

## Conclusions

Under salinity, plants have evolved multifaceted adaptive strategies to cope with the detrimental damages [51]. On one hand, this study identified the morpho-physiologic and ionomic changes in the shoots and roots of rapeseed plants under salinity stress. On the other hand, this study ascertained the core genes, encoding antioxidant enzymes, phytohormones, nutrient transporters and other stress-responsive proteins, among their multicopy family members through the high-resolution expression profiling, which will provide elite and definite gene resources for the molecular breeding of salinity-resistant rapeseed germplasm.

## Methods

### Growth conditions and salt treatments

Considering the rapeseed cultivar of Zhongshuang 11 (a winter cultivar), having well-known information on genome sequences, is an elite genotype with high oil quality, seed production, and strong stress resistance [19], we used Zhongshuang 11 as the rapeseed lines studied in the following experiments. Rapeseed seedlings, which was originally obtained from Prof. Jin-yong Huang (jinyhuang@zzu.edu.cn, Zhengzhou University, Zhengzhou, 450,001, Henan Province, China), were hydroponically grown with Hoagland nutrient solution in an illuminated growth chamber [52]. The cultivated conditions were set according to the report by Hua et al. [52].

For the determination of morpho-physiologic parameters, uniform rapeseed plants after 7-day seed germination were grown under NaCl-free (control) condition for 10 days. After that, the plants were changed to a solution containing 0–250 mM NaCl for 5 days until plant sampling. For the transcriptome sequencing, uniform rapeseed plants after 7-day seed germination were grown for 10 d under NaCl-free condition, and then they were changed to the solution containing 200 mM NaCl for 12 h until sampling.

### Leaf and root system architecture analysis

An LI-COR LI-3100C leaf area meter was used to determine total leaf areas of rapeseed plants. Specific leaf weight was used to assess leaf thickness, and was calculated according to the following equation: specific leaf weight (g FW cm^− 2^) = leaf FW (g) / leaf area (cm^2^) [53].

Roots of rapeseed seedlings were subjected to an image scanner, and then total root length, root volume, root surface area, and root average diameter were analyzed using WinRHIZO Pro (Regent Instruments, QC, Canada).

### Photosynthesis-related parameter analysis

A Li-Cor 6400 photosynthesis system was used to determine the following net photosynthetic rate (*P*_*n*_, μmol m^− 2^ s^− 1^), stomatal conductance (*g*_*s*_, mol H_2_O m^− 2^ s^− 1^), intercellular CO_2_ concentration (*C*_*i*_, μmol mol^− 1^), and transpiration rate (*T*_*r*_, mmol H_2_O m^− 2^ s^− 1^). The *Pn*, g_*s*_, *C*_*i*_, and *T*_*r*_ were measured at the following condition: photon intensity of 1300 μmol m^− 2^ s^− 1^, leaf temperature of 28.0 ± 1.0 °C, relative humidity of 50.0% ± 1%, and atmospheric CO_2_ concentration of 400 ± 5.0 mmol mol^− 1^.

SPAD values of rapeseed leaves were determined using a SPAD-502 chlorophyll meter (Konica Minolta, Tokyo, Japan). Chlorophyll and carotenoid pigments were extracted using 80% isopropyl alcohol (v/v) for 24 h in dark, and then the concentration of purified extract was determined with a UV-1800 spectrophotometer (MAPADA, Shanghai, China) at 663.2, 646.8, and 470 nm. The anthocyanin was isolated using an isolation buffer (CH_3_OH:H_2_O:H_2_O_2_ = 60:13:2, *v*/*v*/*v*). Subsequently, the concentration of the extract was determined with the UV-1800 spectrophotometer at 530 and 657 nm.

### Microscopy analysis

Pieces of rapeseed leaves of approximately 1 mm^2^ were taken to determine intracellular ultrastructures using a transmission electron microscope (H-7650; Hitachi, Tokyo, Japan) [54]. Stomatal density, morphology, and wax coat of the sampled leaf pieces was determined using a scanning electron microscope (JSM-6390/LV, JEOL, Tokyo, Japan) [55]. The leaf samples with at least three independent biological replicates were examined for electron microscopy analysis.

### Ionomic analysis

Over-dried shoot and root tissues were added to a HNO_3_/HClO_4_ mixture (4:1, v/v) at 200 °C until the digestion was completed. The diluted supernatant was determined to quantify the concentrations of mineral elements using an inductively coupled plasma mass spectrometry (ICP-MS; NexIONTM 350X, PerkinElmer).

### Determination of osmoregulatory substances

Concentrations of malondialdehyde (MDA), which was extracted using thiobarbituric acid, were spectrophotometrically assayed at the wavelengths of 450 nm, 532 nm, and 600 nm [50]. Proline concentrations were determined at 520 nm with an ultraviolet spectrophotometer (UV-160, Shimadzu, Tokyo, Japan) using the ninhydrin assay [56].

Soluble protein concentrations were determined using the Bradford reagent, and the absorbance of the sample extract was determined at 595 nm [57]. Concentration of soluble sugars were determined in ethanol extract of rapeseed plants with the anthrone method [58]. Betaine was extracted using 0.375% (w/v) Reinecke salt following the protocol described by [59], and the absorbance was read at 525 nm [60].

### Phytohormone assay

Fresh rapeseed samples were prepared to obtain the phytohormone extract [61]. The standard auxin (indole-3-acetic acid, IAA), cytokinin (CTK), gibberellin (GA), abscisic acid (ABA), and jasmonic acid (JA), and ABA were purchased from Sigma-Aldrich (St. Louis, MO, USA) or OlChemIm (OlChemIm, Olomouc, Czech Republic). The phytohormone concentrations were determined by ultra-fast liquid chromatography-electrospray ionization tandem mass spectrometry (UFLC-ESI-MS) [62].

### ROS determination and enzyme activity assay

Fresh leaves and roots were harvested and immediately frozen. Potassium phosphate buffer (pH 7.8) and 0.1% (w/v) trichloroacetic acid were used to obtain the O_2_^−^ and H_2_O_2_ extracts, respectively. The absorbance of the aforementioned obtained extract was spectrophotometrically determined at the wavelengths of 530 and 390 nm, respectively [63].

The SOD activity was spectrophotometrically determined at 560 nm using the nitroblue tetrazolium method [64]. The POD activity was spectrophotometrically assayed by monitoring the formation of guaiacol at 470 nm [65]. The CAT activity was calculated according to the study by Aebi [66]. The APX activity was assayed according to ascorbate oxidation at 290 nm [67].

### High-throughput transcriptome sequencing

Rapeseed seedlings after 7-day seed germination were grown under NaCl-free conditions for 10 days, and then were transferred to the nutrient solution to which was added to 200 mM NaCl for 12 h until sampling.

Shoots and roots of the rapeseed plants aforementioned were harvested, and three independent biological replicates were used for each treatment. Pre-chilled Trizol (Takara Bio Inc., Kusatsu, Shiga, Japan) was used to isolate total RNA, following which the RNA integrity number (RIN) was assessed. A total of 12 RNA samples with the RIN values > 8.0 were obtained to construct strand-specific cDNA libraries, which were further employed for the paired-end sequencing (read length = 150 bp) on a lane of an Illumina Hiseq 4000 platform. The FPKM values were normalized to quantify the gene expression abundances, and both FDR and *P* values < 0.05 were used to identify the differentially expressed genes (DEGs) [23]. PANTHER (http://www.pantherdb.org/data/) [68] and KEGG (http://www.kegg.jp/) [69], respectively, were used to perform GO and pathway enrichment analysis of the DEGs. Heat maps showing differential gene expression were delineated using a Multiexperiment Viewer (http://www.tm4.org/mev.html) [70].

### Reverse-transcription quantitative polymerase chain reaction assays

Total RNA of each sample was extracted by using pre-chilled TRIzol reagent (Invitrogen, Carlsbad, CA, USA) according to the manufacturer’s recommendations. After treating RNA samples with RNase-free DNase I, total RNA was used as templates for cDNA synthesis with the PrimeScript™ reverse transcription (RT) Reagent Kit with gDNA Eraser (Perfect Real Time; TaKaRa, Shiga, Japan). To detect relative expression of target genes, RT–quantitative polymerase chain reaction (RT-qPCR) assays were conducted under an Applied Biosystems StepOne™ Plus system (Thermo Fisher Scientific, Waltham, MA, USA). The RT-qPCR program was set according to the following thermal cycles: 95 °C for 3 min, 40 cycles of 95 °C for 10 s, and 60 °C for 30 s. Expression levels of the target genes were normalized using two public house-keeping genes, *BnaEF1-α* [71] and *BnaGDI1* [72], based on the 2^-ΔΔC^_*T*_ method [73]. The RT-qPCR primers used in this study are listed in Supplementary Table S1.

### Statistical analysis

The Statistical Productions and Service Solutions 17.0 (SPSS, Chicago, IL, USA) was used to perform statistical testes. One-way analysis of variance followed by Tukey’s honestly significant difference (*P* < 0.05, *P* < 0.01, and *P* < 0.001) multiple comparison tests was used to determine the significance differences.

## Supplementary Information

**Additional file 1:** **Table S1.**

**Additional file 2:** **Supplementary Figure S1.** *Pearson* correlation coefficients of the RNA-seq data between each pair of biological replicates. Note: C, control; T, treatment (200 mM NaCl); S, shoot; R, root.

## Data Availability

All the data and materials that are required to reproduce these findings can be shared by contacting the corresponding author, Dr. Ying-peng Hua (yingpenghua@zzu.edu.cn). The raw data of transcriptome sequencing have submitted to the National Centre for Biotechnology Information (NCBI) (http://www.ncbi.nlm.nih.gov/) with the Bioproject of PRJNA340053.

## Abbreviations

ABA: Abscisic acid
AKT: Arabidopsis K^+^ transport system
AtKC1: *Arabidopsis thaliana* K^+^ channel
APX: Ascorbate peroxidase
BASS: Bile acid: Na^+^ symporter family protein
CAT: Catalase
CHX: Cation/H^+^ exchanger
CNBD: Cyclic nucleotide binding domain
CPA: Cation/H^+^ antiporter
CTK: Cytokinin
DEG: Differentially expressed gene
GA: Gibberellic acid
GORK: Gated outwardly-rectifying K^+^ channel
GS/GOGAT: Glutamine synthetase/glutamine oxoglutarate aminotransferase
HAK: High-affinity K^+^ transporter
IAA: Indoleacetic acid
JA: Jasmonic acid
KAT: K^+^ channel in *Arabidopsis thaliana*
KEA: K^+^ efflux antiporter
KT: K^+^ transporter
KUP: K^+^ uptake transporter
MDA: Malondialdehyde
NHX: Na^+^/H^+^ exchanger
POD: Peroxidase
RT-qPCR: Reverse transcription quantitative polymerase chain reaction
SKOR: Stellar K^+^ outward rectifier
SOD: Superoxide dismutase
SOS: Salt overly sensitive
