# Supplementary material for: Comprehensive dissection into morpho-physiologic responses, ionomic homeostasis, and transcriptomic profiling reveals the systematic resistance of allotetraploid rapeseed to salinity
Source: BMC Plant Biol. 2020 Nov 24;20:534. doi: 10.1186/s12870-020-02734-4 (PMC7685620; doi:10.1186/s12870-020-02734-4)
Supplement: Supplementary file 1 — Additional file 1: Table S1. [file 12870_2020_2734_MOESM1_ESM.docx]

Supplementary Table S1 Overview of the genome-wide mRNA sequencing data

| Sample name | | Clean reads | Clean bases | Error rate (%) | Q20 (%) | Q30 (%) | GC content (%) | Total mapped | Uniquely mapped |
| --- | --- | --- | --- | --- | --- | --- | --- | --- | --- |
| CR^a^ | CR_1 | 49127920 | 7191115019 | 0.0237 | 98.6 | 95.4 | 48.0 | 90.2% | 84.4% |
|  | CR_2 | 47927964 | 7001212434 | 0.0238 | 98.6 | 95.4 | 48.0 | 89.8% | 83.6% |
|  | CR_3 | 56458508 | 8294688903 | 0.0238 | 98.6 | 95.4 | 47.8 | 90.3% | 84.6% |
| CS | CS_1 | 54963384 | 8080151751 | 0.0231 | 98.8 | 96.0 | 48.8 | 91.5% | 84.4% |
|  | CS_2 | 57913436 | 8260697011 | 0.0230 | 98.9 | 96.1 | 48.2 | 90.6% | 82.7% |
|  | CS_3 | 52638188 | 7776891873 | 0.0235 | 98.7 | 95.6 | 48.7 | 92.2% | 86.0% |
| TR | TR_1 | 57780656 | 8507431258 | 0.0234 | 98.7 | 95.8 | 47.9 | 90.3% | 83.8% |
|  | TR_2 | 54183370 | 7971795942 | 0.0233 | 98.7 | 95.8 | 47.9 | 91.0% | 85.7% |
|  | TR_3 | 54228392 | 7977474443 | 0.0233 | 98.8 | 95.9 | 48.0 | 91.1% | 85.7% |
| TS^b^ | TS_1 | 55499170 | 8144620009 | 0.0233 | 98.8 | 95.8 | 48.1 | 92.2% | 85.7% |
|  | TS_2 | 58903104 | 8651375343 | 0.0235 | 98.7 | 95.6 | 48.3 | 92.3% | 86.1% |
|  | TS_3 | 49425686 | 7262445400 | 0.0233 | 98.8 | 95.8 | 48.1 | 92.3% | 85.8% |
| Mean | | 54087482 | 7926658282 | 0.0234 | 98.7 | 95.7 | 48.1 | 91.1% | 84.9% |
| Total | | 649049778 | 95119899386 |  | | | | | |

^a, b^Note: C, control; T, treatment (NaCl); S, shoot; R, root.
