# Supplementary material for: Comprehensive dissection into morpho-physiologic responses, ionomic homeostasis, and transcriptomic profiling reveals the systematic resistance of allotetraploid rapeseed to salinity
Source: BMC Plant Biol. 2020 Nov 24;20:534. doi: 10.1186/s12870-020-02734-4 (PMC7685620; doi:10.1186/s12870-020-02734-4)
Supplement: Supplementary file 2 — Additional file 2: Supplementary Figure S1. Pearson correlation coefficients of the RNA-seq data between each pair of biological replicates. Note: C, control; T, treatment (200 mM NaCl); S, shoot; R, root. [file 12870_2020_2734_MOESM2_ESM.docx]

**Supplementary Figures**


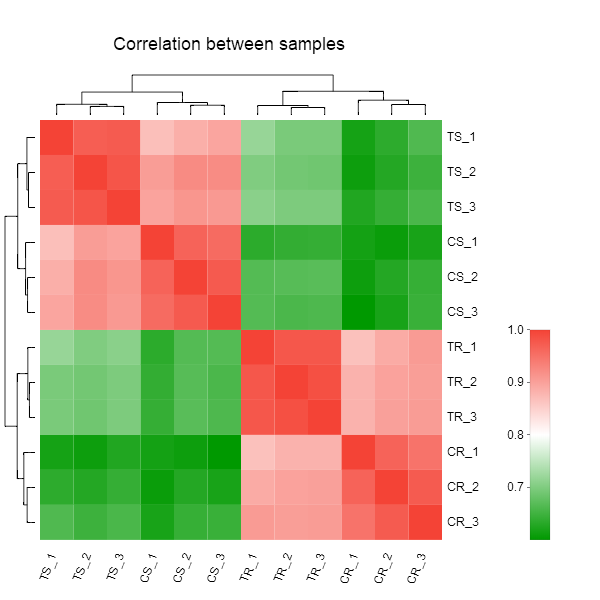


**Supplementary Figure S1** *Pearson* correlation coefficients of the RNA-seq data between each pair of biological replicates. Note: C, control; T, treatment (200 mM NaCl); S, shoot; R, root.
